# Correction: Assessing the exchange coupling in binuclear lanthanide(iii) complexes and the slow relaxation of the magnetization in the antiferromagnetically coupled Dy_2_ derivative

**DOI:** 10.1039/c5sc90037a

**Published:** 2015-06-26

**Authors:** Chun Y. Chow, Hélène Bolvin, Victoria E. Campbell, Régis Guillot, Jeff W. Kampf, Wolfgang Wernsdorfer, Frédéric Gendron, Jochen Autschbach, Vincent L. Pecoraro, Talal Mallah

**Affiliations:** a Department of Chemistry , University of Michigan , Ann Arbor , Michigan 48108 , USA . Email: vlpec@umich.edu; b Laboratiore de Chimie et Physique Quantiques , Université Toulouse III, 118 route de Narbonne , 31062 Toulouse , France; c Institut de Chimie Moléculaire et des Matériaux d'Orsay , CNRS , Université de Paris Sud 11 , 91405 Orsay Cedex , France . Email: talal.mallah@u-psud.fr; d Institut Néel , CNRS , Université J. Fourier , BP 166 25, Avenue des Martyrs , 38405 Grenoble , France; e Department of Chemistry , University at Buffalo , State University of New York , Buffalo , NY 14260-3000 , USA

## Abstract

Correction for ‘Assessing the exchange coupling in binuclear lanthanide(iii) complexes and the slow relaxation of the magnetization in the antiferromagnetically coupled Dy_2_ derivative’ by Chun Y. Chow *et al.*, *Chem. Sci.*, 2015, **6**, 4148–4159.



## 

A grant from the European Community's Seventh Framework Programme was inadvertently omitted in the Acknowledgements of this manuscript. The following information should be added to the Acknowledgements section:

“The research leading to these results has received funding from the European Community's Seventh Framework Programme (FP7/2007–2013) under grant agreement number 611488.”

The Royal Society of Chemistry apologises for these errors and any consequent inconvenience to authors and readers.

